# Characterization of linkage disequilibrium, consistency of gametic phase and admixture in Australian and Canadian goats

**DOI:** 10.1186/s12863-015-0220-1

**Published:** 2015-06-25

**Authors:** Luiz F. Brito, Mohsen Jafarikia, Daniela A. Grossi, James W. Kijas, Laercio R. Porto-Neto, Ricardo V. Ventura, Mehdi Salgorzaei, Flavio S. Schenkel

**Affiliations:** Centre for Genetic Improvement of Livestock, University of Guelph, Guelph, ON Canada; Canadian Centre for Swine Improvement Inc, Ottawa, ON Canada; CSIRO Agriculture Flagship, Brisbane, QLD Australia; Beef Improvement Opportunities, Guelph, ON Canada; The Semex Alliance, Guelph, ON Canada

**Keywords:** Effective population size, Genomic selection, Goat breeds, Goat 50 k panel, LD

## Abstract

**Background:**

Basic understanding of linkage disequilibrium (LD) and population structure, as well as the consistency of gametic phase across breeds is crucial for genome-wide association studies and successful implementation of genomic selection. However, it is still limited in goats. Therefore, the objectives of this research were: (i) to estimate genome-wide levels of LD in goat breeds using data generated with the Illumina Goat SNP50 BeadChip; (ii) to study the consistency of gametic phase across breeds in order to evaluate the possible use of a multi-breed training population for genomic selection and (iii) develop insights concerning the population history of goat breeds.

**Results:**

Average r^2^ between adjacent SNP pairs ranged from 0.28 to 0.11 for Boer and Rangeland populations. At the average distance between adjacent SNPs in the current 50 k SNP panel (~0.06 Mb), the breeds LaMancha, Nubian, Toggenburg and Boer exceeded or approached the level of linkage disequilibrium that is useful (r^2^ > 0.2) for genomic predictions. In all breeds LD decayed rapidly with increasing inter-marker distance. The estimated correlations for all the breed pairs, except Canadian and Australian Boer populations, were lower than 0.70 for all marker distances greater than 0.02 Mb. These results are not high enough to encourage the pooling of breeds in a single training population for genomic selection. The admixture analysis shows that some breeds have distinct genotypes based on SNP50 genotypes, such as the Boer, Cashmere and Nubian populations. The other groups share higher genome proportions with each other, indicating higher admixture and a more diverse genetic composition.

**Conclusions:**

This work presents results of a diverse collection of breeds, which are of great interest for the implementation of genomic selection in goats. The LD results indicate that, with a large enough training population, genomic selection could potentially be implemented within breed with the current 50 k panel, but some breeds might benefit from a denser panel. For multi-breed genomic evaluation, a denser SNP panel also seems to be required.

**Electronic supplementary material:**

The online version of this article (doi:10.1186/s12863-015-0220-1) contains supplementary material, which is available to authorized users.

## Background

Goats are highly adaptable to different environmental conditions being raised all over the world for milk, meat and fibre production. Although they present reasonable reproductive and productive performance, it is necessary to improve their production efficiency to become more competitive with other livestock industries. In this regard, genetic selection plays a very important role and substantial genetic gain has been achieved using traditional breeding methods. However, there are some important traits that are difficult or expensive to measure (e.g. resistance to diseases, carcass traits, etc.), measured late in life or sex limited (e.g. milk production and composition). The development of genomic technologies means that new methods have become available such as genomic selection (GS) proposed by Meuwissen et al. [1].

GS has been successfully implemented in dairy cattle breeding programs and it is either under development or in the process of being implemented in other animal species. In dairy cattle the main advantage of GS is that it reduces the generation interval increasing the genetic gain per year. In goats, the generation interval is relatively lower than cattle, but could still be reduced. GS could also help to increase the selection intensity, which would increase productivity and reduce costs in breeding programs. As a first step for goat breeders, a 50 K SNP panel [2] has been developed by the International Goat Genome Consortium (IGGC), facilitating both genome wide association studies (GWAS) and the opportunity to implement GS.

One relevant parameter to the implementation of genomic selection in a breeding program is the extent to which linkage disequilibrium (LD) persists across the genome and how it varies between populations. LD is defined as a non-random association of alleles at two or more loci and is influenced by population history breeding system and the pattern of geographic subdivision [3]. The marker density required for successful GWAS and subsequently genomic selection, depends on the extent of LD across the genome [4]. A low LD level would require a higher marker density to enable markers to capture most of the genetic variation in the population. The persistence of LD has been evaluated in a number of domesticated animal species including pigs [5–7], horses [8], cattle [9–11] and sheep [12, 13]. A preliminary evaluation has also been conducted in goats using French dairy breeds [14]. Given the persistence of LD varies considerably between breeds in other species [13], it is important to characterise LD in a diverse collection of goat populations.

In addition to linkage disequilibrium accuracy of genomic selection also depends on the number of records available to estimate marker effects (training population). This may be a limitation factor for implementation of GS in goats because the genotyping costs are still relatively high compared to the economic value of the animals. An alternative to increase the number of animals in the training population is combining data from multi-breed populations. To obtain good accuracies of predictions using multi-breed populations it is required not only high LD between the markers and the quantitative trait loci (QTL) in each breed, but also high consistency of gametic phase between the markers and the QTL across breeds. Consistency of gametic phase is a measure of the degree of agreement of gametic phase for pairs of markers between two populations [6] that is also dependent of the difference on allele frequencies and relatedness between the two populations.

A variety of evolutionary phenomena impact observed allele frequencies distributions and the persistence of linkage disequilibrium. These include forces such as genetic drift migration, natural selection, and mutation rate. Therefore, population history strongly influences the extent of LD, particularly in domestic animal populations which have undergone bottlenecks during both domestication and the subsequent formation of breeds. The strength of these forces is likely to be different across the farm yard animal species, and indeed between breeds within each species. This prompted the investigation, in this study, of aspects of population history including ancestral effective population size, which can be inferred from the observed extent of LD [15–17].

There are many goat breeds been raised commercially all over the world and during the years they were characterized by high levels of admixture followed by animal movement. For instance goats were carried by the early explorers to America and Oceania [18] and some African breeds were also introduced more recently, such as South African Boer [19]. In order to better understand how modern goat breeds developed historically and to what degree they may have been mixed in the past, one alternative is to look at their breed composition through an analysis of admixture and/or principal components analysis (PCA).

Basic understanding of LD and population structure as well as the consistency of gametic phase across breeds is crucial for the implementation of genomic selection and is still limited in goats. Therefore, the objectives of this research were to estimate genome-wide levels of LD in Australian and Canadian goat breeds using data generated with the Illumina Goat SNP50 BeadChip to study the consistency of gametic phase between different breeds in order to evaluate the possible use of a multi-breed training population for genomic selection and develop insights concerning the population history of goat breeds.

## Methods

The Canadian animals included in this study were managed in accordance with the Recommended Code of Practice for the Care and Handling of Farm Animals - GOATS (Canadian Agri - Food Research Council) [20]. All the samples were collected from commercial farms and the animal owners agreed to be involved in the project through their respective associations i.e. Ontario Goat and Société des éleveurs de chèvres laitières de race du Quebec. Samples were collected by well trained staff following industry best practices. Animal handling and sample collection from Australian animals were performed in accordance with Animal Ethics, CSIRO Brisbane Animal Ethics Committee.

### Animals

The data analyzed in this study included genotypes of goats raised for milk meat and fibre production from two sources: i) a set of 976 Canadian goats from six breeds (Alpine, Boer, LaMancha, Nubian, Saanen and Toggenburg) and ii) 175 Australian goats from three breeds (Boer, Cashmere and Rangeland). The total number of genotyped animals for each breed by country is described in Table 1. The Canadian animals were from 25 commercial herds located in the provinces of Ontario and Quebec, two artificial insemination (AI) centres, and the Agriculture and Agri-Food Canada (AAFC) Centre for Animal Genetic Resources (Saskatoon, Saskatchewan). Most of the samples were ear notches (76 %), but also included extracted DNA samples from older animals (13 %), blood samples (9 %) and semen straws (2 %).

**Table 1:** Number of animals and amount of SNPs excluded during the quality control procedure of the genotype data

Breed		Excluded SNPs*					Remaining SNPs
	N	MAF < 0.05	SNP CR < 0.90	HWE	Het.	Total^3^	
AL^1^	403	3,828	2,358	39	1	6,820	45,268
SA^1^	318	4,155	2,358	45	2	7,140	44,946
		MAF < 0.15	SNP CR < 0.90	HWE	Het.	Total^3^	
LN^1^	81	10,321	2,358	54	156	13,436	38,650
NU^1^	54	16,013	2,379	21	242	19,233	32,853
TO^1^	53	15,388	2,362	63	870	19,233	32,863
BO^1^	67	13,282	2,374	17	185	16,438	35,648
BO^2^	61	11,562	55	81	471	15,029	37,057
CA^2^	48	9,508	147	33	689	13,272	38,814
RL^2^	66	4,695	57	52	274	7,980	44,106

^1^Canada, ^2^Australia, ^3^It was excluded 2,958 SNPs without chromosome number and/or position information or SNPs located in the sexual chromosomes. AL: Alpine, SA: Saanen, LN: LaMancha, NU: Nubian, TO: Toggenburg, BO: Boer, CA: Cashmere, RL: Rangeland; *some SNPs were excluded due to more than one criterion,, MAF = minor allele frequency; CR = call rate; HWE = *χ* ^2^ test for Hardy-Weinberg equilibrium (*p*-value < 10^−6^). Het: excess of heterozigosity (>0.15)

The Australian populations and the genotypes derived from them have been described previously [21]. In brief animals were sampled from three different regions: 61 Boer goats from the Yarrabee goat herd in Queensland, 66 Rangeland goats from outback New South Wales and 48 Cashmere goats from Queensland. DNA was extracted from whole blood using the Qiagen Blood and Tissue extraction kit following the manufacturer’s instructions.

### SNP genotyping and data filters

All the animals were genotyped using the Illumina goat SNP50 BeadChip (Illumina Inc. San Diego, CA) containing 53,347 single nucleotide polymorphisms (SNPs). SNP filtering and quality control conducted on the Australian populations resulted in analysis of a final marker set containing 52,088 loci [21]. The Canadian and Australian datasets were merged and only the 52,088 SNPs present in both datasets were kept for further analysis.

The genotyping quality control was performed within breed to remove SNPs and/or samples that could bias the LD estimates. SNPs with MAF lower than 5 % (for Alpine and Saanen breeds) or 15 % (for other breeds which have a much smaller number of genotyped animals) were removed prior to estimation of LD to prevent monomorphic loci inflating LD. SNPs were also excluded if the call rate was lower than 90 %, if they deviated significantly from Hardy–Weinberg equilibrium (HWE, *p* < 10^−6^) or if they presented a heterozygosity excess (>0.15, [22]). Only mapped autosomal SNPs were included for further analyses. Missing SNP genotypes were not imputed due to the limited number of genotyped animals in each breed. Besides the SNPs quality control, we also performed a quality control to animals, where individuals that had SNP call rate < 0.90 were removed. The number of SNPs excluded during the quality control procedure by each criterion is presented in Table 1. The number of SNPs per breed remaining after exclusions ranged from 32,853 to 45,268 out of 52,088 SNPs.

### Extent of linkage disequilibrium

The extent of LD between markers was measured using r^2^ as proposed by Hill and Robertson [23], which is the squared correlation between alleles at two loci. It can be expressed as:

$$ {r}^2=\frac{D^2}{f(A)f(a)f(B)f(b)} $$

where *D* = *f* (*AB*) – *f* (*A*) *f* (*B*) and *f* (*AB*), *f* (*A*), *f* (*a*), *f* (*B*), and *f* (*b*), are observed frequencies of haplotype AB and alleles A, a, B, and b, respectively. However, the number of animals genotyped for this study was not enough to reconstruct haplotypes accurately. Thus, a D estimate suggested by Lynch and Walsh [24] was used:

$$ D=\frac{N}{N-1}\left[\frac{4{N}_{AABB}+2\left({N}_{AABb}+{N}_{AaBB}\right)+{N}_{AaBb}}{2N}-2\times f(A)\times f(B)\right] $$

where *N* is the total number of animals, and *N*_*AABB*_, *N*_*AABb*_, *N*_*AaBB*_, and *N*_*AaBb*_ are the corresponding number of individuals in each genotypic category (AABB, AABb, AaBB, and AaBb). Another commonly used pair-wise measure of LD is D’ [25]. The reason for using r^2^ rather than D’ is that r^2^ is less sensitive to allele frequency and small sample size [26]. Values range from 0 (no LD) to 1 (complete LD) between two markers. If we consider the r^2^ between a bi-allelic marker and an (unobserved) bi-allelic QTL, r^2^ is the proportion of variation caused by the alleles at a QTL that is explained by the markers [27].

We calculated r^2^ for each pair of loci on each chromosome to determine the LD between adjacent SNPs, and the LD decay over different distances. To examine the decay of LD with physical distance, SNP pairs on the autosomes were sorted into bins based on pair-wise marker distance and the average of each bin was calculated. We defined 20 distance bins: lower than 0.02 Mb, from 0.02 until 0.1 defined every 0.01 Mb from 0.1 to 1 Mb defined every 0.1 Mb from 1 to 1.2 Mb and greater than 1.2 Mb.

### Consistency of gametic phase

The consistency of gametic phase was defined by the Pearson correlation of signed r values between two breeds. For each marker pair with a measure of r^2^ the signed r value was determined by taking the square root of the r^2^ value and assigning the appropriate sign based on the calculated disequilibrium (D) value. Data was sorted into bins based on pair-wise marker distance to determine the breakdown in the consistency of gametic phase across distances and to assess the consistency of gametic phase at the smallest distances possible, given the number of genotyped SNPs. For each distance bin, the signed r values were then correlated between all 36 pairs of breeds using the CORR procedure in SAS (SAS Institute Inc., Cary, USA).

### Ancestral effective population size

The r^2^ measures combined with markers distance can be used to estimate the approximate effective population size (N_e_) at a given point in the past time. The N_e_ in each generation was determined based on the expectation of r^2^ in different distances and assuming a model without mutation as described by Sved [15]: $E\left({r}^2\right)=\frac{1}{1+4{N}_ec}$, in which, *c* is the distance in Morgans between the SNPs. N_e_ is the effective population size and r^2^ is the average r^2^ value at a given distance. Each genetic distance (c) corresponds to a value of *t* generations in the past. This value was calculated as *t* = 1/(2c) as suggested by Hayes et al. [17].

The ancestral N_e_ was investigated at 21 time points from 5 until 1500 generations in the past. The distances (*c*) were taken as the middle of a range and the average r^2^ value was estimated at that distance. N_e_ was then calculated at each distance using that specific average r^2^.

### Admixture analysis

In order to have an insight about the evolutionary history of the breeds included in this study we performed an admixture analysis. The same genotype quality control presented in Table 1 was applied to the admixture analysis. We used the ADMIXTURE software [28] to determine the level of admixture of each animal. This software applies a model based on a clustering algorithm that identifies subgroups that have distinctive allele frequencies. It places individuals into *k* predefined clusters.

The choice of an appropriate value for *k* is a notoriously difficult statistical problem. It seems that this choice should be guided by knowledge of a population’s history [28]. In this study we evaluated *k* from 6 to 10 as it would be a more representative value of the expected number of subpopulations in our data set. Two out of nine populations were from the same breed (Australian and Canadian Boer populations). Furthermore, it is known that the Rangeland is a composite breed population. So only results for *k* = 7 were shown, which have a more reasonable biological interpretation, as suggested by Pritchard et al. [29].

### Principal component analysis (PCA)

In order to better assess the breed composition of the animals and for graphically display the results we also performed a principal component analysis. Principal components were calculated from the genomic relationship matrix (**G**) using prcomp function of R [30]. The **G** matrix was calculated using the method described by VanRaden [31]:

$$ \boldsymbol{G} = \frac{\left(\boldsymbol{M}-2\boldsymbol{P}\right)\left(\boldsymbol{M}-2\boldsymbol{P}\right)\boldsymbol{\hbox{'}}}{2{\displaystyle \sum }{\boldsymbol{p}}_{\boldsymbol{i}}\left(1-{\boldsymbol{p}}_{\boldsymbol{i}}\right)}, $$

where **M** is a matrix of counts of the alleles “A” (with dimensions equal to the number of animals by number of SNPs), **p**_**i**_ is the frequency of allele “A” of the i^th^ SNP, **P** is a matrix (with dimensions equal to the number of animals by number of SNPs) with each row containing the **p**_**i**_ values, **I** is the identity matrix (of size equal to the number of animals). Missing values in **M** were replaced by 2 times the frequency of allele “A” in the breed.

## Results

### SNP frequency and distribution

**Fig. 1:**
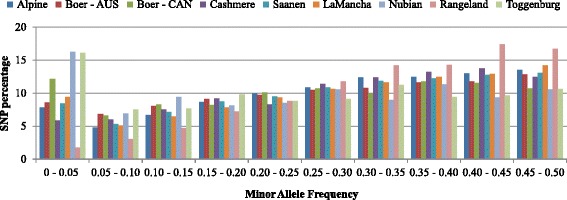
Distribution of SNPs by MAF ranges and breed. AUS: Australia, CAN: Canada

The level of genetic diversity present within and between the goat populations can be measured by the number of polymorphic loci and their allele frequencies distributions. Table 1 indicates that the Rangeland Alpine and Saanen breeds had the highest number of loci remaining after filtering based on MAF, HWE and other metrics. Fig. 1 presents the distribution of SNP by MAF range, and shows that Rangeland goats had the highest rate of high MAF loci, where more than 90 % of SNPs displayed MAF in excess of 0.15. Conversely, the Nubian and Toggenburg breeds had 67.41 and 68.68 % of loci with MAF > 0.15. Only one animal from the Rangeland breed was excluded due to low call rate (<0.90). Alpine and Saanen breeds presented very similar SNPs distribution for all MAF ranges. Canadian Boer population presented a higher proportion of SNPs with MAF < 0.15 compared to the Australian Boer population.

A descriptive summary of chromosomes and SNPs for the Alpine breed (larger sample size) is shown in Table 2. Diploid cells of *Capra hircus* contain 29 homologous autosomal pairs (CHI) and one pair of sex chromosomes. The total autosomal genome length was 2402.526 Mb with the shortest CHI being 41.478 Mb (CHI25) and the longest CHI being 154.929 Mb (CHI1).

**Table 2:** Summary of analyzed single nucleotide polymorphism (SNP) markers for each *Capra hircus* autosome (CHI) for the Alpine breed

CHI	Length (Mb)	SNP (n)	Mean distance (Mb)	Longest gap (Mb)	Mean r^2^
1	154.929	2,939	0.0527 ± 0.0273	0.2724	0.156
2	135.409	2,578	0.0525 ± 0.0292	0.3905	0.142
3	116.773	2,129	0.0548 ± 0.0296	0.2931	0.138
4	115.925	2,192	0.0529 ± 0.0296	0.4332	0.151
5	110.992	2,049	0.0540 ± 0.0316	0.4313	0.133
6	114.319	2,176	0.0525 ± 0.0290	0.3011	0.145
7	106.469	1,972	0.0540 ± 0.0304	0.3245	0.160
8	110.894	2,134	0.0519 ± 0.0264	0.2591	0.146
9	90.267	1,725	0.0523 ± 0.0262	0.2640	0.135
10	99.104	1,894	0.0522 ± 0.0264	0.2678	0.169
11	105.280	1,964	0.0535 ± 0.0284	0.2998	0.143
12	83.588	1,545	0.0541 ± 0.0344	0.7093	0.160
13	80.605	1,491	0.0540 ± 0.0319	0.3693	0.149
14	92.298	1,717	0.0537 ± 0.0300	0.2954	0.164
15	78.973	1,504	0.0525 ± 0.0298	0.4343	0.139
16	77.625	1,468	0.0528 ± 0.0267	0.2214	0.153
17	71.841	1,334	0.0537 ± 0.0322	0.4750	0.135
18	60.990	1,161	0.0524 ± 0.0291	0.3044	0.149
19	62.124	1,124	0.0553 ± 0.0312	0.3173	0.116
20	71.198	1,341	0.0530 ± 0.0267	0.2238	0.144
21	66.714	1,307	0.0510 ± 0.0253	0.2654	0.137
22	57.863	1,076	0.0537 ± 0.0303	0.3036	0.142
23	49.385	950	0.0519 ± 0.0257	0.2341	0.140
24	61.693	1,211	0.0508 ± 0.0269	0.2536	0.135
25	41.478	787	0.0526 ± 0.0296	0.2421	0.127
26	50.153	941	0.0531 ± 0.0287	0.2997	0.139
27	44.099	840	0.0524 ± 0.0302	0.3827	0.142
28	43.204	841	0.0513 ± 0.0273	0.2589	0.120
29	48.332	878	0.0550 ± 0.0331	0.4650	0.122
Overall	2,402.526	45,268	0.0530	0.7093	0.144

After application of quality control filters to remove low quality data the 50 k SNP panel showed good coverage of the genome with an average gap size between adjacent SNP varying from 0.05 to 0.07 Mb. Additional file 1: Tables S1.a and S1.b shows the largest intervals by chromosome and breed. The largest gaps were observed on CHI12 (0.7093 Mb), CHI17 (1.1399 Mb), CHI3 (1.9366 Mb), CHI12 (0.6780 Mb), CHI12 (0.7093 Mb), CHI7 (1.6214 Mb), CHI22 (1.0613 Mb), CHI29 (0.4990 Mb), CHI25 (1.1201 Mb) for Alpine, Boer (Australian population), Boer (Canadian population), Cashmere, Saanen, LaMancha, Nubian, Rangeland and Toggenburg animals, respectively. The chromosomes that presented larger gaps in most breeds were: CHI12, CHI17 and CHI29. Most of the breeds with a smaller number of animals had the largest average gap size between adjacent SNPs, due to the exclusion of SNPs with minor allele frequency (MAF) lower than 0.15, while for Alpine and Saanen breeds, MAF threshold was 0.05. However, for the Rangeland breed, even considering a MAF threshold of 0.15, the number of excluded SNPs was similar with those from Alpine and Saanen breeds.

Additional file 2: Table S2 presents the distribution of SNPs by chromosome for each breed. The greater range in the number of SNPs/Mb was observed for the Boer breed (Australian population) from 13.62 (CHI13) to 17.31 (CHI28) SNPs/Mb and the shorter range was observed for the Alpine breed and it varied from 18.09 (CHI19) to 19.63 (CHI19) SNPs/Mb.

### Extent of linkage disequilibrium within goat breeds

Linkage disequilibrium was estimated separately within each of 9 goat populations using r^2^. The average linkage disequilibrium (r^2^) between adjacent SNPs by breed and average distance between adjacent SNPs (Mb) are presented in Table 3. Average r^2^ between adjacent SNP pairs was highest within the two geographically distinct populations of Boer goats (0.287 and 0.289) and lowest for the Rangeland and Alpine populations (0.110 and 0.144). The average r^2^ appears to reflect breed diversity whereby genetically diverse populations have generally lower average LD between adjacent loci. LD was also compared between chromosomes, revealing some variation (Additional file 3). The chromosomes that presented higher levels of LD were not the same for most breeds, except for Canadian and Australian Boer populations that presented more similar LD estimates.

**Table 3:** Average linkage disequilibrium (r^2^) and average distance (Mb) between adjacent SNPs by breed

Breed	r^2^	Distance (Mb)
Alpine^1^	0.144	0.0530
Saanen^1^	0.153	0.0534
LaMancha^1^	0.194	0.0621
Nubian^1^	0.276	0.0733
Toggenburg^1^	0.248	0.0731
Boer^1^	0.287	0.0674
Boer^2^	0.289	0.0648
Cashmere^2^	0.182	0.0620
Rangeland^2^	0.110	0.0545

^1^Canada; ^2^Australia

**Fig. 2:**
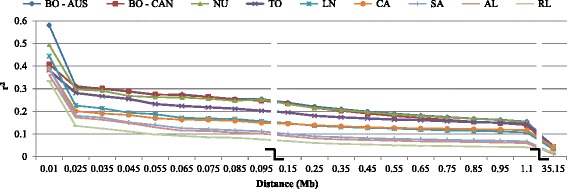
Average r^2^ values at given distances (Mb) for Australian and Canadian goats. BO-AUS: Australia Boer, BO-CAN: Canadian Boer, NU: Nubian, TO: Toggenburg, LN: LaMancha, CA: Cashmere, SA: Saanen, AL: Alpine, RL: Rangeland

LD is expected to decline as the recombination and physical distance between the markers increases. Fig. 2 displays the average LD values at given distance ranges for each breed (see also Additional file 4). High LD values were observed only at small distances between pairs of SNPs. For all the breeds LD decays rapidly as distance between the two SNPs increases. The average r^2^ estimates for the Rangeland population were the lowest values across all distances. It was followed by Alpine and Saanen. Alpine and Saanen breeds showed similar pattern of LD, which could be explained by their common ancestral origin [14].

It is important to note that the number of animals varied between groups (Table 1) and this has the potential to influence the observed LD. A correction for sampling error was applied that accounts for the number of haplotypes observed per population. Corrected r^2^ was calculated as (r^2^ – 1/N)/(1 – 1/N), where N is the number of haplotypes or twice the number of individuals [23]. The Additional file 5 presents the estimated and corrected r^2^ values for all the populations included in this study. However, the differences were small and all the results presented in this paper are based on non-corrected r^2^ estimates.

Boer (Australian and Canadian populations) and Nubian had the highest levels of LD across all distances. The r^2^ values for Canadian and Australian Boer animals were very similar for short distances bins except for distances up to 0.02 Mb. The r^2^ similarities could be indicating that they were managed together until few generations ago (around 5 generations ago). The Australian Boer goats presented higher estimates at long distances compared to Canadian Boer goats.

Trends across distances were very similar (Fig. 2) for all breeds and the LD level decayed at a very similar rate. The extent of LD decreased substantially from the first (up to 0.02 Mb) to the second range of distances (between 0.02 and 0.03 Mb). The number of SNP pairs at distances < 0.02 Mb was quite small though, which is also indicated by the high standard deviation values (Additional file 4: Tables S4.a and S4.b). The mean r^2^ decreased more slowly with increasing distance. LD levels were smaller than 0.05 at distances greater than 1.20 Mb for all breeds. The low level of long range LD may indicate that these breeds have not been under intense selection or have had large effective population size in the recent past.

### Admixture and principal components analyses

Breed composition for each animal was calculated using the admixture model as described by Alexander et al. [28]. This determines the proportion of a given genome originated from each of *k* ancestral clusters defined as seven in this study. Fig. 7 and Table 4 show the proportion of each cluster, averaged across individuals within population. Some breeds have distinct genotypes (less clusters) based on SNP50 genotypes, such as the Boer, Cashmere and Nubian populations. The other groups share higher genome proportions with each other, indicating higher admixture and a more diverse genetic composition. The Rangeland population contains the highest rate of admixture, consistent with it being an unmanaged feral population founded by mixing of a number of breeds [21]. The admixture analysis presented here indicates contributions of mainly Cashmere, Nubian and Boer breeds into the Rangeland population. However, there is also a contribution from some dairy breeds. On average, around 13, 27 and 50 % of the Rangeland goat genome was in common to that found in Nubian, Boer and Cashmere, respectively (Table 4). LaMancha breed presents a contribution of Alpine and Nubian breeds (8 and 5 %, respectively). Saanen breed shares a higher proportion of the genome with Alpine (12 %), followed by Toggenburg (6 %) and LaMancha (4 %). The Saanen and Alpine breeds were managed together until few decades ago. However, the Saanen breed appears to be more mixed compared to Alpine breed. Australian and Canadian Boer populations were grouped together with an average of 95 % of their genome in common.

**Table 4:** Average breed composition of 9 goat populations given 7 clusters estimated by ADMIXTURE software

Breed^1^	Cluster 1	Cluster 2	Cluster 3	Cluster 4	Cluster 5	Cluster 6	Cluster 7
Alpine^1^	0.8689 ± 0.1250	0.0109 ± 0.0314	0.0383 ± 0.0445	0.0160 ± 0.0203	0.0222 ± 0.0214	0.0080 ± 0.0102	0.0354 ± 0.0446
Saanen^1^	0.1169 ± 0.1324	0.0069 ± 0.0239	0.7603 ± 0.1967	0.0145 ± 0.0182	0.0411 ± 0.0326	0.0040 ± 0.0079	0.0561 ± 0.0536
LaMancha^1^	0.0755 ± 0.0573	0.0516 ± 0.0421	0.0344 ± 0.0682	0.0192 ± 0.0174	0.7880 ± 0.1666	0.0042 ± 0.0062	0.0269 ± 0.0211
Toggenburg^1^	0.0767 ± 0.1389	0.0049 ± 0.0126	0.0256 ± 0.0391	0.0103 ± 0.0169	0.0193 ± 0.0300	0.0027 ± 0.0049	0.8601 ± 0.2218
Nubian^1^	0.0033 ± 0.0072	0.9546 ± 0.0724	0.0026 ± 0.0046	0.0141 ± 0.0198	0.0093 ± 0.0131	0.0132 ± 0.0438	0.0027 ± 0.0048
Boer^1^	0.0029 ± 0.0125	0.0204 ± 0.0630	0.0011 ± 0.0036	0.0214 ± 0.0205	0.0021 ± 0.0040	0.9506 ± 0.0719	0.0011 ± 0.0028
Boer^2^	0.0014 ± 0.0041	0.0032 ± 0.0069	0.0039 ± 0.0071	0.0189 ± 0.0256	0.0187 ± 0.0226	0.9481 ± 0.0443	0.0054 ± 0.0114
Rangeland^2^	0.0289 ± 0.0204	0.1337 ± 0.0279	0.0276 ± 0.0188	0.4971 ± 0.1015	0.0218 ± 0.0161	0.2704 ± 0.1509	0.0203 ± 0.0115
Cashmere^2^	0.0023 ± 0.0064	0.0164 ± 0.0171	0.0054 ± 0.0098	0.9539 ± 0.0519	0.0046 ± 0.0071	0.0121 ± 0.0165	0.0050 ± 0.0085

^1^Canada; ^2^Australia

Figure 8 presents the first and second principal components calculated based on the **G** matrix. It shows that some breeds present clear clusters while others are genetically closer to each other. Australian and Canadian Boer were clustered together. The dairy breeds were clustered apart from the dual purpose/fibre/meat breeds. The Rangeland animals were clustered close to Nubian, Cashmere and Boer, what was also observed in the admixture analysis.

### Linkage phase

**Fig. 3:**
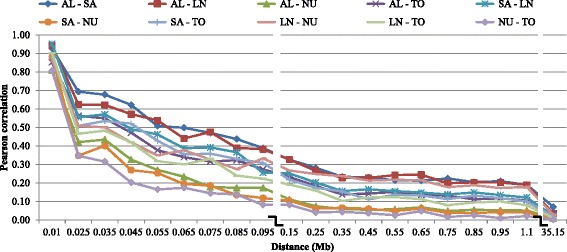
Consistency of gametic phase (Pearson correlations of signed r values) at given distances for 10 selected breed pairs. AL: Alpine, LN: LaMancha, NU: Nubian, SA: Saanen and TO: Toggenburg

**Fig. 4:**
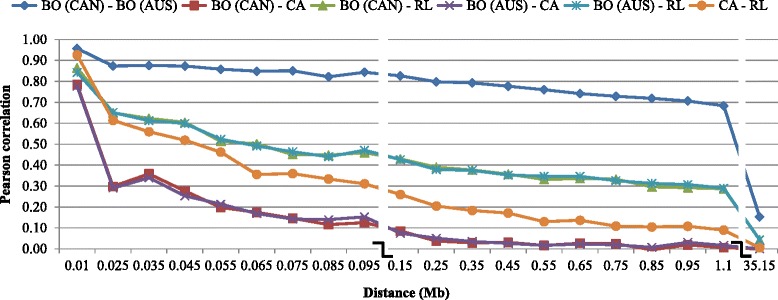
Consistency of gametic phase (Pearson correlations of signed r values) at given distances for 6 selected breed pairs. BO (AUS): Australia Boer, BO (CAN): Canadian Boer, CA: Cashmere and RL: Rangeland

The strength of consistency in gametic phase between breeds has implications for the design of successful genomic prediction programs. Specifically it influences what (if any) breed combinations can be merged to form a single training set to estimate SNP effects. Figs. 3 and 4 present the consistency of gametic phase (Pearson correlation between signed r values) between some breed pairs, while Table 5 presents the Pearson correlations between gametic phase of all breeds over distances smaller than 0.20 Mb (above diagonal) and between 0.02 and 0.03 Mb (below diagonal). The estimates for other distances were not presented as they were small. However, it is shown in the Additional file 6 for all distances and breed pairs. The highest consistency of gametic phase was found between Australian and Canadian Boer. This is expected given the two geographically distinct populations were drawn from the same breed. Other groups that presented higher correlations were: Alpine and Saanen, Alpine and LaMancha, Canadian Boer and Rangeland, Australian Boer and Rangeland, and Cashmere and Rangeland. The estimated correlations for all the breed pairs except Canadian and Australian Boer, were lower than 0.70 for all distances greater than 0.02 Mb. The correlations between Australian and Canadian Boer and Cashmere or Rangeland were very similar, suggesting a high relatedness between Australian and Canadian Boer populations.

**Table 5:** Pearson correlations between gametic phase of all breeds for the distances pairs smaller than 0.2 Mb (above diagonal) and between 0.02 and 0.03 Mb (below diagonal)

	Alpine^1^	Saanen^1^	LaMancha^1^	Toggenburg^1^	Nubian^1^	Boer^1^	Boer^2^	Cashmere^2^	Rangeland^2^
Alpine^1^		0.93	0.94	0.85	0.81	0.75	0.74	0.83	0.87
Saanen^1^	0.69		0.95	0.88	0.88	0.79	0.78	0.84	0.87
LaMancha^1^	0.62	0.56		0.90	0.87	0.74	0.76	0.82	0.82
Toggenburg^1^	0.56	0.51	0.47		0.81	0.70	0.75	0.78	0.77
Nubian^1^	0.42	0.35	0.51	0.35		0.76	0.80	0.87	0.88
Boer^1^	0.35	0.36	0.41	0.29	0.34		0.96	0.78	0.86
Boer^2^	0.38	0.38	0.44	0.34	0.32	0.87		0.78	0.84
Cashmere^2^	0.42	0.37	0.38	0.33	0.36	0.30	0.29		0.93
Rangeland^2^	0.50	0.45	0.50	0.39	0.45	0.65	0.65	0.61	

^1^Canada; ^2^Australia

### Ancestral effective population size estimations

**Fig. 5:**
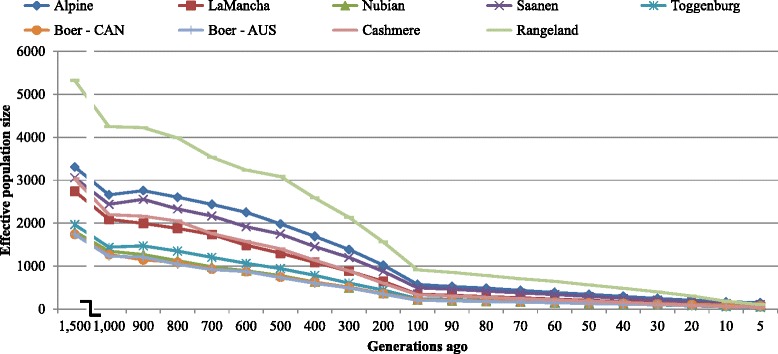
Past effective population size (Ne) over generations based on linkage disequilibrium calculations from 29 autosomes. AUS: Australia, CAN: Canada

**Fig. 6:**
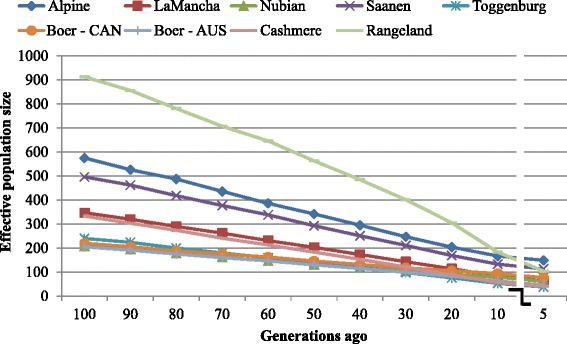
Past effective population size (Ne) from 100 to 5 generations ago based on linkage disequilibrium calculations from 29 autosomes. AUS: Australia, CAN: Canada

A graphical representation of the N_e_ values at each time point from 1500 to five generations ago is given in Figs. 5 and 6. Looking at the N_e_ in the distant past (1500 generations ago), effective populations were found to be ~ 5325, 3309, 3057, 3030, 2742, 1967, 1803, 1743, and 1741 animals for Rangeland Alpine, Saanen, Cashmere, LaMancha, Toggenburg, Nubian, Australian Boer and Canadian Boer populations, respectively. It corresponds to the closest measured time to the goat domestication, which occurred around 10,000 years ago [32]. Based on an average generation interval of 4 years [33], they would have been domesticated around 2500 generations ago. However, there were no enough SNP pairs to accurately estimate N_e_ for more than 1500 generations ago.

The results suggest that N_e_ has been lower in the recent past compared to the ancient past. The effective population size at five generations ago is calculated to be 104, 149, 113, 41, 62, 38, 61, 46 and 77 for Rangeland, Alpine, Saanen, Cashmere, LaMancha, Toggenburg, Nubian, Australian Boer and Canadian Boer populations, respectively. At the most recent measure of effective population size, five generations ago, Alpine breed presented the highest N_e_, followed by Saanen and Rangeland. On the other hand, Toggenburg, Cashmere and Australian Boer populations presented the lowest values. The estimates for Australian and Canadian Boer populations were very similar for most of the measured time, except for the most recent generations studied. The Canadian Boer population presented higher N_e_ than Australian Boer population and it was particularly low at the most recent generations studied.

## Discussion

### Genotypic data and levels of LD

For breeds with small number of samples higher MAF threshold was applied and therefore more SNPs were excluded (Table 1). However, for the Rangeland population, even using a 0.15 MAF threshold, it presented a number of excluded SNPs by MAF criteria similar with Alpine and Saanen breeds, indicating high levels of polymorphism in that population. The high diversity level in the Rangeland population was previously discussed by Kijas et al. [21]. From the breeds included in this study, only Alpine, Boer and Saanen were represented within the SNP discovery panel used during the development of the goat SNP50 chip. Even though, all of them presented high levels of polymorphisms. The observed levels of MAF within breeds should provide enough variability for genomic studies such as genome-wide association studies and genomic evaluations.

The amount of SNPs remaining for the Alpine and Saanen breeds were slightly smaller than those attained by Carillier et al. [14] for the same breeds. They applied a call rate threshold of 98 % a MAF greater than 0.01 and Hardy-Weinberg equilibrium test (p-value < 10^−6^) and validated 46,959 out of 53,347 SNPs. Mucha et al. [34] working with a crossbred population (Alpine, Saanen and Toggenburg), filtered out SNPs that were not in Hard Weinberg equilibrium, had MAF below 0.05, call rate below 0.95 or GC content below 0.6. After the quality control 47,306 markers were available for further analyses. Despite of the number of SNPs excluded in our study, the 50 k panel showed good coverage of the genome.

The number of SNPs excluded due to low SNP call rate (CR) was very similar for the Canadian breeds. The smaller number of SNPs excluded due to low CR for the Australian breeds is due to the pre quality control that was done previously in the Australian dataset in which 1145 markers with call rates lower than 90 % were removed. Greater gaps were observed between SNP pairs in some chromosomes for most of the breeds (e.g. CHI12, CHI17 and CHI29), suggesting that in future development of another SNP panel for goats more SNPs could be included in those chromosomes for better coverage.

In the present study the number of genotyped animals differed considerably across breeds, with the largest number of genotyped animals in Alpine (403) and Saanen (318) breeds (Table 1). The differences in average r^2^ values for the breeds may be in part due to sampling effects, the low numbers of animals genotyped in some breeds and it could be due to different effective population sizes of those populations, which seems particularly appropriate for some breeds. Bohmanova et al. [26] recommended that for Holstein cattle at least 55 animals should be used to avoid overestimation of r^2^. In this study for three populations (Cashmere, Nubian and Toggenburg) there were fewer animals genotyped than that value. To address this concern, we applied a correction suggested by Hill and Robertson [23]. However, even for the Cashmere breed (smallest sample size) the highest difference between r^2^ estimated and corrected were around 0.01 units. Therefore, we decided to present the non-corrected values in the main text.

The average LD estimates in the goat breeds studied were quite variable. For Alpine and Saanen breeds average r^2^ values at 50 kb were slightly smaller than the values reported by Carillier et al. [14] (0.17 at 50 kb). In a crossbred population (Alpine, Saanen, and Toggenburg) Mucha et al. [34] observed a mean r^2^ at 50 kb of 0.18. For the other breeds, this was the first study done, which did not allow us to compare the results.

For the breeds Alpine Cashmere, Saanen, and Rangeland, the LD levels appear to be lower than that reported in Holstein dairy cattle (from 0.18 to 0.31, [35, 9, 36, 10]) or pigs (0.36 to 0.46, [6, 5]). The r^2^ estimates for the Saanen breed were similar with those attained for the Churra breed sheep of 0.152 from 40 to 60 kb [12]. Kijas et al. [13] found average r^2^ values for five sheep breeds for marker pairs at 70 kb apart varying from 0.08 to 0.22.

There is variation in the published extent of LD because the estimates of LD strongly depend on various factors such as: history and structure of the studied population (evolutionary forces that affected the population) sample size, marker type (microsatellites or SNPs), density and distribution of markers, type of method used for haplotype reconstruction, strictness of SNP filtering (threshold of minor allele frequencies and Hardy-Weinberg equilibrium), use of maternal haplotypes only or both maternal and paternal haplotypes [26].

As pointed out in Hayes et al. [17] LD at small distances reflects N_e_ in the distant past whereas LD at large distances reflects N_e_ in the recent past. The r^2^ values for Canadian and Australian Boer animals were very similar for short distances, except for distances up to 0.02 Mb, which could be indicating that they were managed together until few generations ago. The differences observed for distances up to 0.02 Mb could be explained by the small number of SNP pairs used to estimate r^2^ for that distance range. The higher r^2^ estimates at long distances observed in Australian Boer goats compared to the Canadian Boer population could be due to a smaller effective population size in the more recent past in the Australian Boer population compared to the Canadian one or it could be due to the fact that all Australian Boer animals were sampled in the same region and they could be more related than the average of the Australian Boer population. The standard deviations values (SD) for the r^2^ estimates at given distances (Additional file 4) were quite high, mainly for shorter distances, which may be due to the smaller number of SNP pairs available for the r^2^ estimations.

The extent of LD decreased substantially from the first (up to 0.02 Mb) to the second range of distances (between 0.02 and 0.03 Mb) (Fig. 2). The low level of long range LD may indicate that these breeds have not been under intense selection or genetic drift.

Alpine and Saanen were the breeds with the largest sample sizes. The higher observed levels of LD at short ranges in some of the other breeds could be due to sampling but they are more likely to be due to smaller effective population size in those breeds, as Rangeland population also presented low r^2^ values. Therefore, it would be interesting to confirm the LD results obtained in this investigation using a larger number of genotyped animals.

A higher level of LD is related to a higher accuracy of genomic estimated breeding values. Some studies (e.g. [1, 37]) have recommended that an r^2^ value greater than 0.2 would be sufficient for genomic selection. At the average distance between adjacent SNPs in the goat 50 k SNP panel (~0.06 Mb) the breeds LaMancha, Nubian, Toggenburg, and Australian and Canadian Boer exceeded or approached this value. This indicates that, with a large enough training population, genomic selection could potentially be implemented with reasonable accuracy using the current 50 k panel within breed, but the other breeds might benefit from a denser panel. For the Rangeland population, the LD levels were very low even for short distances, suggesting that this breed come from a highly heterogeneous population and a higher density panel might be needed to implement genomic selection in this breed.

### Admixture and principal component analyses and linkage phase

**Fig. 7:**
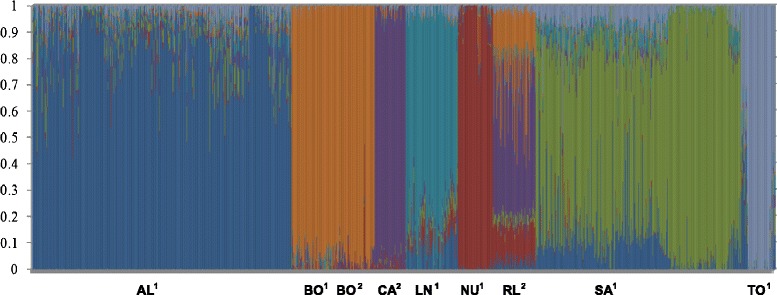
Breed composition per animal using ADMIXTURE software. ^1^: Canada, ^2^:Australia, AL = Alpine, BO = Boer, CA = Cashmere, LN = LaMancha, NU = Nubian, RL = Rangeland, SA = Saanen and TO = Toggenburg

The results show that a great number of animals have a significant portion of their genotype coming from another cluster (Fig. 7). Boer, Cashmere and Nubian breeds seem to have a smaller level of admixture compared to the other breeds, indicating that there is less remaining from any other ancestral breed that may have interacted with them.

For the animals that had estimates of breed composition more diverse (less than 75 % of their genes coming from a single breed) it can be assumed that a more recent admixture event could have occurred. According to Larmer et al. [38] this may be useful in identifying locations of certain QTL that are present in only one breed. If an animal has a phenotype that is significantly different from other animals in the breed to which it is registered and chunks coming from other breeds can be identified in the genome we could propose that one or more of those chunks have a QTL for that trait on them.

**Fig. 8:**
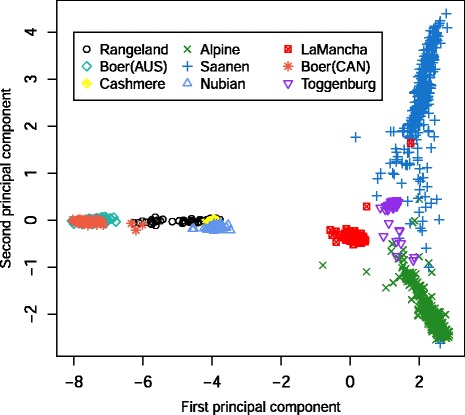
Plot of the first 2 principal components of the genomic relationship matrix for all animals, colored by breed

The higher level of admixture seen in the Saanen breed when compared to the Alpine breed implies that a greater degree of admixture has undergone since these breeds diverged historically. Consistently PCA (Fig. 8) also showed this trend. Animals from Alpine and Saanen breeds showed more spread clusters, indicating a higher breed admixture level among those breeds and other dairy breeds such as LaMancha and Toggenburg. LaMancha and Toggenburg showed clear individual clusters and a smaller genetic variation among animals from within those breeds. The larger degree of admixture seen in the Rangeland population is consistent with its evolutionary history, as the Rangeland goats are largely unmanaged feral goats. The results indicate that Boer, Cashmere and Nubian breeds are likely to have contributed to create the feral population. On average, 50 % of the Rangeland genome was in common to that found in the Cashmere breed. It indicates that the Rangeland population may have been formed by the introgression of mainly Boer and Nubian animals in the Cashmere genetics to develop the Rangeland population. PCA (Fig. 8) also confirmed this relationship, where animals from Nubian, Cashmere, Boer and Rangeland were closely clustered compared to the dairy breeds. This sharing of the gene pool may be due to mixing of the breeds as discussed before, especially for Rangeland population.

Canadian and Australian Boer populations seem to share a great proportion of the genome. The small level of admixture coming from other breeds (clusters) indicates that admixture may have taken place on average, in the distant past. The high degree of genotype sharing among both Boer populations is consistent with their evolutionary history, as the Boer breed was developed in South Africa [39] and exported to Canada and Australia a few decades ago. Furthermore, according to Casey and Van Niekerk [39], the Boer breed was formed with infusion of Indian and European blood, which could explain the admixture contribution, even small, from other breeds. The close relationship between Australian and Canadian Boer populations was also confirmed in the PCA plot (Fig. 8), where animals were clustered together based on the first two principal components.

The PCA analysis showed that the Illumina 50 K goat beadchip was able to discriminate most of the breeds. Some of them were more clearly clustered while others were clustered more closely. However this trend is consistent with the breeds history. Huson et al. [40] reported that the Illumina 50 K goat beadchip can effectively distinguish goat populations, specifically indigenous African goat populations. In a comparison of 14 African goat breeds, New Zealand Boer, three Italian Alpine breeds, and six United States of America breeds, the first principal component generated a continental categorization by Italy, United States of America, and Africa with the second principal component distinguishing the Boer breed.

The consistency of gametic phase between breeds indicates whether or not different breeds could potentially be pooled into one common training population to better estimate SNP effects. For goat genomic evaluation this would be very important due to the fact that there is a small number of genotyped animals in the breeds with small population size. The highest consistency of phase was found between Australian and Canadian Boer populations suggesting a greater level of relatedness between these populations. They may be still connected through exchange of genetic material or have diverged a few generations ago. It was also confirmed in the admixture analysis, where they were always grouped together. According to Malan [19], Boer goats were imported to North America directly from South Africa or via Australia or New Zealand, which is another evidence of their close relationship. The correlation values for them were consistent until greater distances, indicating that both populations could be pooled in a single training population. The other groups that presented higher correlations were: Alpine and Saanen, Alpine and LaMancha, Canadian Boer and Rangeland, Australian Boer and Rangeland and Cashmere and Rangeland. Based on the admixture levels observed for some breeds, it was expected a higher consistency of phase among them. However, even for those breed pairs the consistency of gametic phase between adjacent markers was not high enough to support the pooling of breeds in a training population for genomic selection. The estimated correlations for all the breed pairs, except Canadian and Australian Boer, were lower than 0.70 for distances greater than 0.02 Mb. It indicates that markers and QTL phases might not be strongly associated across those breeds.

Carillier et al. [14] found consistency of gametic phases at 50 kb (i.e. average distance between two SNPs) among French Alpine and Saanen breeds of 0.56. According to them, the two goat breeds (Alpine and Saanen) were genetically close until a couple of generations ago. In dairy cattle, de Roos et al. [41] evaluated the effect of combining multiple populations on the reliability of genomic predictions and concluded that the benefits of combining populations in a training set were higher when the populations have diverged for only a few generations ago, when the marker density was high, and when heritability was low. From the simulation studies reported by these authors, populations that had diverged six generations ago presented a correlation of phase higher than 0.8 for distances up to 0.45 Mb. Therefore, for multi-breed genomic evaluation in goats, a denser SNP panel seems to be required. For implementing genomic selection using the 50 k panel in goat breeds, other ways to increase the training population should be sought, such as genotyping more animals in each breed or collaborate with other countries and share genotypes and phenotypes/EBVs for genomic selection.

### Ancestral effective population size

We observed an initial pattern of decreasing N_e_ with values of over 1740 for Australian and Canadian Boer populations and 5325 for Rangeland population estimated in the distant past (1500 generations ago) and values closer or even smaller than 100 estimated at 5 generations ago (Figs. 5 and 6 and Additional file 7).

The N_e_ estimates at 5 generations ago found for Alpine and Saanen breeds (149 and 113 respectively) were similar with those reported by Larroque et al. [42] for French Alpine and Saanen breeds, 143 and 120, respectively. Garcia-Gamez et al. [12] have reported a N_e_ estimate of 128 in the more recent generation studied for Churra breed sheep population. Alpine and Saanen breeds are the most common dairy breeds raised over the world, which is reflected by their highest N_e_ measures in the most recent time. The similar estimates attained for both Boer populations are another evidence of their relatedness. The differences observed in the most recent past may be due to sampling errors or smaller number of animals in the Canadian population compared to the South African population, where Australian and Canadian Boer animals were probably imported. The high N_e_ observed in the ancient past for the Rangeland population reflects the great level of admixture observed for this breed. As observed in the admixture analysis, Boer, Cashmere, Nubian and other breeds contributed to its formation.

According to Meuwissen [43] a threshold of N_e_ = 100 would be necessary to ensure that an animal population is long-term viable in terms of genetic diversity. Our results of current effective population size are above the threshold only for 3 breeds, indicating that care should be taken in this regard to ensure that the effective population size and consequently a reasonable diversity level are maintained.

## Conclusions

At the average distance between adjacent SNPs in the current 50 k SNP panel (~0.06 Mb) the breeds LaMancha, Nubian, Toggenburg and Australian and Canadian Boer exceeded or approached the level of linkage disequilibrium that is useful (r^2^ > 0.2) for genomic prediction. This indicates that, with a large enough training population, genomic selection could potentially be implemented within breed with the current 50 k panel, but the breeds Alpine, Saanen, Cashmere and Rangeland might benefit from a denser panel.

The highest consistency of gametic phase was found between Australian and Canadian Boer populations indicating a greater level of relatedness between these two breeds and a possibility of pooling them in a single reference population. However, for the other breeds, the consistency of gametic phase between adjacent markers is not high enough to encourage the pooling of breeds in a single training population for genomic selection. For multi-breed genomic evaluation, a denser SNP panel seems to be required. Therefore, other ways to increase the training population for genomic selection using the 50 k panel should be sought, such as genotyping more animals in each breed and/or collaborating with other countries for sharing genotypes and phenotypes/EBVs.

## Additional files

Additional file 1: Table S1. Largest gaps between adjacent SNPs by chromosome and breed. Showing the largest intervals between adjacent SNPs for each autosome and for all 9 goat populations. Additional file 2: Table S2. Number of SNPs/Mb by breed for each autosome (CHI). Presenting the distribution of SNPs by chromosome for each breed. Additional file 3: Table S3. Linkage disequilibrium (r^2^) estimates by chromosome for each breed. Showing the LD estimates by chromosome for each breed. Additional file 4: Table S4. Average r^2^ values (± standard deviation) at a given distance range. Displays the average LD values at given distance ranges for each breed. Additional file 5: Table S5. Average r^2^ and corrected r^2^ at given distances. Presenting the estimated and corrected r^2^ values for all the populations included in this study. Additional file 6: Table S6. Pearson correlations between gametic phase of all breeds pairs. Showing the estimates for the Pearson correlations between gametic phase of all breeds pairs, including those there were not presented in the main text. Additional file 7:Table S7. Effective population size for all studied breeds for a given number of generations ago. Presenting the effective population size for all studied breeds for a given number of generations ago estimated based on the LD levels.

## Abbreviations

AAFC: Agriculture and Agri-Food Canada
AI: Artificial insemination
AL: Alpine breed
AUS: Australia
BO: Boer breed
CA: Cashmere breed
CAN: Canada
Chr: Chromosome
CHI: *Capra hircus* homologous autosomal pairs
CR: Call Rate
CSIRO: Commonwealth Scientific and Industrial Research Organisation
DNA: Deoxyribonucleic acid
EBV: Estimated Breeding value
GEBV: Genomic Estimated Breeding Value
GS: Genomic Selection
GWAS: Genome-Wide Association Studies
HWE: Hardy-Weinberg Equilibrium
IGGC: International Goat Genome Consortium
kb: kilo base pairs
LD: Linkage Disequilibrium
LN: LaMancha breed
MAF: Minor Allele Frequency
Mb: Mega base pairs
N_e_: Effective population size
NU: Nubian breed
PCA: Principal component analysis
QTL: Quantitative Trait Loci
RL: Rangeland population
SA: Saanen breed
SD: Standard deviation
SNP: Single Nucleotide Polymorphism
TO: Toggenburg breed
